# *PURA*-Related Neurodevelopmental Disorder: Insight from Eight New Cases

**DOI:** 10.3390/genes17070765

**Published:** 2026-06-30

**Authors:** Agnieszka Madej-Pilarczyk, Marzena Gawlik, Beata Chałupczyńska, Jagoda Błaszkiewicz, Dorota Wicher, Agata Cieślikowska, Anila Babameto-Laku, Krystyna Chrzanowska, Elżbieta Ciara

**Affiliations:** 1 Department of Medical Genetics, The Children’s Memorial Health Institute, Member of the European Reference Network ITHACA, 04-730 Warsaw, Poland; a.madej-pilarczyk@ipczd.pl (A.M.-P.); m.gawlik@ipczd.pl (M.G.); b.chalupczynska@ipczd.pl (B.C.); j.blaszkiewicz@ipczd.pl (J.B.); d.wicher@ipczd.pl (D.W.); a.cieslikowska@ipczd.pl (A.C.); k.chrzanowska@ipczd.pl (K.C.); 2 Service of Genetics Laboratory, Faculty of Medicine, University Hospital Center “Mother Teresa”, 1000 Tirana, Albania; laku62@yahoo.com

**Keywords:** epileptic encephalopathy, molecular genetics, *PURA* gene variants, *PURA*-related neurodevelopmental disorder, *PURA*-NDD, rare disorder, hypotonia

## Abstract

**Background:** *PURA*-related neurodevelopmental disorder (*PURA*-NDD; OMIM #616158) is a rare autosomal dominant condition caused by pathogenic variants in the *PURA* gene encoding Purα, a multifunctional protein involved in DNA replication, transcriptional regulation, and RNA transport. Since its initial description in 2014, *PURA*-NDD has been increasingly recognized as a distinct clinical entity with early onset and a broad phenotypic spectrum. The clinical presentation is characterized primarily by neonatal hypotonia, global developmental delay, intellectual disability, feeding difficulties, and epilepsy, along with additional features such as respiratory insufficiency, movement disorders, hypersomnolence, and variable dysmorphic traits. Despite a relatively recognizable core phenotype, marked inter-individual variability often limits the ability to establish a definitive clinical diagnosis based on phenotype alone. This underscores the essential role of molecular genetic testing in the differential diagnosis of rare neurodevelopmental disorders. **Patients and methods:** We report a cohort of eight individuals (four males and four females) aged 17 months to 15.5 years with *PURA*-related neurodevelopmental disorder (*PURA*-NDD), evaluated using a genotype-first diagnostic strategy supported by comprehensive genomic testing, including next-generation sequencing (NGS) panels and whole-genome sequencing (WGS). Patients were referred for the evaluation of nonspecific neurodevelopmental features, including neonatal hypotonia, respiratory distress, and epilepsy, in the absence of a definitive clinical diagnosis. **Results:** Molecular analysis identified eight heterozygous variants in *PURA*, of which four (50%) were novel: c.311T>G p.(Met104Arg), c.406_407del p.(Gln136Glyfs64), c.515A>C p.(Gln172Pro), and c.885delinsGC p.(His296Profs21). The remaining variants included previously reported missense and frameshift changes associated with *PURA*-NDD, as well as one variant previously reported in ClinVar. **Conclusions:** Our findings not only confirm the core clinical features of *PURA*-related neurodevelopmental disorder but also contribute to a more comprehensive delineation of its phenotypic spectrum. The detailed characterization of our cohort broadens the range of recognized clinical manifestations and further highlights the marked phenotypic heterogeneity of *PURA*-NDD. In addition, the identification of both novel and previously reported pathogenic variants expands the mutational spectrum of *PURA* and underscores the importance of integrating clinical, molecular, and bioinformatic data for accurate variant interpretation. Although genotype–phenotype correlations remain incompletely understood, emerging evidence suggests potential associations between variant type or location and clinical severity, warranting further investigation in larger cohorts. The recognition of characteristic neonatal features may facilitate earlier diagnosis and implementation of supportive multidisciplinary management. Overall, this study illustrates how genomic technologies not only improve diagnostic yield in rare disorders but also refine disease definition, enhance the understanding of underlying pathogenic mechanisms, and support the development of more precise genotype–phenotype correlations. Further studies involving larger cohorts and long-term follow-up are needed to better define the full clinical and molecular spectrum of *PURA*-NDD.

## 1. Introduction

PURA syndrome (*PURA*-related neurodevelopmental disorder; *PURA*-NDD; OMIM #616158) is a rare autosomal dominant neurodevelopmental disorder caused by pathogenic variants in the *PURA* gene. The genes encode Purine-rich element-binding protein A (Purα), a highly conserved multifunctional protein involved in DNA replication, transcriptional regulation, and RNA transport [1,2]. Purα includes the N-terminal glycine-rich region, three motifs (PUR I–III), and a C-terminal glutamine–glutamate-rich domain [3].

*PURA*-NDD represents a paradigmatic example of how advances in molecular genetics, particularly next-generation sequencing (NGS) technologies, have transformed the discovery, delineation, and diagnostic classification of rare neurodevelopmental disorders. Since the first independent reports linking *PURA* variants to human disease in 2014, the syndrome has been increasingly recognized as a distinct clinical entity associated with early-onset neurological impairment [4,5,6,7]. To date, more than 700 patients have been described in the literature. The estimated prevalence of the syndrome is 1 in 1 million; however, this figure may be underestimated due to differences in the availability of genetic testing across regions [8].

Clinically, PURA syndrome is characterized by neonatal hypotonia, global developmental delay, intellectual disability, feeding difficulties, and epilepsy [6,7,8,9,10]. Additional manifestations may include respiratory insufficiency, movement disorders, hypersomnolence, and variable dysmorphic features [10,11,12,13,14,15]. Despite a recognizable core phenotype, substantial inter-individual variability has been reported, ranging from severe neonatal encephalopathy to milder developmental presentations [12,16,17].

To date, the majority of pathogenic *PURA* variants have arisen de novo and include missense, nonsense, frameshift, and splice-site alterations distributed throughout the coding region [5,6,12,18,19]. Initial studies suggested limited evidence for clear genotype–phenotype correlations; however, more recent cohort-based analyses have proposed potential associations between variant class or localization and specific clinical manifestations, including seizure prevalence, motor development, and cognitive outcomes. Protein-truncating variants in the *PURA* gene are more often associated with speech deficits in comparison to missense or in-frame variants. Variants resulting in the deletion of the entire *PURA* gene (del5q31.3) or of several PUR-rich domains are associated with a higher frequency of respiratory problems, walking disability, or speech deficits than variants affecting a single PUR motif [12,18,19]. Nevertheless, these findings remain inconclusive, largely due to small cohort sizes, heterogeneous data collection, and variability in clinical reporting [9,12,18]. The available evidence suggests that pathogenic *PURA* variants appear to be fully penetrant. To date, there are no reports of clinically unaffected individuals with pathogenic *PURA* variants.

Given these limitations, systematic genotype–phenotype studies in well-characterized cohorts are essential to better define the clinical spectrum of PURA syndrome and to identify meaningful correlations between genetic variation and disease presentation [11,12,18,19]. Such efforts may improve diagnostic accuracy, inform prognosis, and contribute to the development of clinical management strategies [20]. Furthermore, a deeper understanding of variant-specific effects may provide insights into the molecular mechanisms underlying *PURA*-related neurodevelopmental dysfunction [21].

In this study, we present a cohort-based genotype–phenotype analysis of eight individuals with molecularly confirmed PURA syndrome. We aim to (i) further delineate the phenotypic spectrum of PURA syndrome, (ii) explore potential genotype–phenotype correlations, and (iii) contribute to the improved clinical and molecular characterization of this emerging disorder.

## 2. Materials and Methods

### 2.1. Patients

This study included eight unrelated individuals with *PURA*-related neurodevelopmental disorder (*PURA*-NDD), comprising four males and four females, aged 17 months to 15.5 years. Seven patients were referred for clinical evaluation and molecular genetic testing at the Children’s Memorial Health Institute (CMHI) in Warsaw, Poland, between 2017 and 2025 and one patient (Patient 3) was referred to the Service of Genetics Laboratory, Faculty of Medicine, University Hospital Center “Mother Teresa”, Tirana, Albania. Structured clinical information was collected using a standardized form completed by the referring clinician or extracted from the medical records.

The study population represented patients with nonspecific neurodevelopmental phenotypes evaluated through a reverse phenotyping approach and cases in which molecular findings ultimately guided the recognition of the syndrome. Written informed consent for genetic testing and study participation was obtained from all participants or their legal guardians.

### 2.2. Genetic Analysis

Molecular analyses were conducted for eight individuals using genomic DNA automatically isolated from peripheral blood leukocytes with either the MagNA Pure LC 2.0 (Roche Diagnostics, Risch-Rotkreuz, Switzerland) or the MagCore Nucleic Acid Extractor HF16Plus (RBC Bioscience, New Taipei City, Taiwan), in accordance with the manufacturers’ instructions. The DNA samples were quantified using the Qubit dsDNA HS Assay Kit (Life Technologies, Eugene, OR, USA), and integrity was evaluated by electrophoresis on a 1% agarose gel. Approximately 50 ng of high-quality genomic DNA was subjected to next-generation sequencing (NGS). Sequencing was performed using in-house CMHI NGS panel comprising over 1000 clinically relevant genes (Roche Diagnostics, Risch-Rotkreuz, Switzerland; Twist Bioscience, San Francisco, CA, USA) (Supplementary Table S1). The CMHI panel used in this study is a custom-designed, diagnostic tool developed specifically for first-tier genetic screening of pediatric patients suspected of having monogenic disorders caused by pathogenic variants in genes that are major contributors to these diseases. The panel covers the entire mitochondrial genome (mtDNA) and the coding regions of 1345 selected disease-associated nuclear genes, together with the canonical splice-site regions (±10 nucleotides of adjacent intronic sequences). Deep intronic and regulatory regions were not included in the panel design.

In one case (P3), the whole genome sequencing (WGS) was performed using a NEBNext Ultra II DNA Library Prep Kit (Illumina, San Diego, CA, USA) and involved extracting high-quality genomic DNA, fragmenting it to ~200–500 bp, followed by end repair and A-tailing to prepare fragments for adapter ligation. Enriched libraries were sequenced in paired-end mode (2 × 100 bp) on the HiSeq 1500 or NovaSeq 6000 platforms (Illumina, San Diego, CA, USA), following the manufacturer’s protocols. The mean sequencing depth was 130×, with more than 95% of the targeted regions achieving a minimum coverage of 20×. Raw FASTQ reads were aligned to the human reference genome (GRCh38/hg38). Variant detection was carried out using several open-source algorithms, including GATK HaplotypeCaller, MuTect2, FreeBayes, and DeepVariant, to enhance sensitivity for the identification of single-nucleotide variants (SNVs) and small insertions/deletions (indels). Copy number variants (CNVs) were assessed with CNVkit and Decon. Sequence alignments were inspected using Integrative Genomics Viewer (IGV), version 2.16.2 [22]. Performance of the variant-calling pipeline was evaluated using the following quality metrics: sensitivity of 99.5%, precision exceeding 99%, and an F-score above 99%. These values are based on experimental validation of the applied analytical workflow. Variant annotation was conducted using the Ensembl Variant Effect Predictor (VEP), followed by comprehensive characterization through multiple data sources. These included the following: (1) Population frequency databases, such as the Genome Aggregation Database (gnomAD v4.1.0) and an in-house Polish database (POLdb) comprising over 24,000 individuals evaluated for suspected rare genetic disorders. (2) In silico predictions of functional impact on protein structure and function, incorporating both ensemble machine learning-based scores (e.g., BayesDel, REVEL) and individual predictive tools (including AlphaMissense, CADD, EIGEN, FATHMM-MKL, MutationTaster, PolyPhen-2, and SIFT). Variants affecting canonical splice regions were additionally assessed using splicing prediction tools such as SpliceAI, ADA, MaxEntScan, Pangolin, and RF. (3) Reference variant databases, including ClinVar, the Leiden Open Variation Database (LOVD), and the Human Gene Mutation Database (HGMD) [23]. Variant classification was performed in accordance with the recommendations of the American College of Medical Genetics and Genomics and the Association for Molecular Pathology (ACMG/AMP), as well as the Association for Clinical Genomic Science (ACGS) [24,25]. Variants categorized as benign or likely benign were excluded from further analysis, and only pathogenic (P), likely pathogenic (LP), and variants of uncertain significance (VUS) were retained and subsequently correlated with the clinical phenotype. Parental samples, when available, were analyzed using NGS trio approaches or targeted Sanger sequencing with the BigDye Terminator v3.1 Kit (Applied Biosystems, Waltham, MA, USA) on an ABI 3130 Genetic Analyzer (Applied Biosystems, Waltham, MA, USA), following the manufacturer’s instructions. Analyses were performed to determine inheritance patterns and confirm de novo status, whereas cases without parental material were classified as having unknown inheritance patterns. The nomenclature of reported molecular variants follows the Human Genome Variation Society (HGVS) guidelines using the MANE Select human *PURA* reference sequence, NM_005859.5 (for cDNA) and NP_005850.1 (for protein).

## 3. Results

### 3.1. Clinical Characteristics of Patients

The study cohort consisted of eight probands: four males and four females with a diagnosis of *PURA*-related neurodevelopmental disorder, established after wide genetic testing and a reverse phenotyping approach. Detailed clinical characteristics of the cohort are presented in Table 1. All patients (8/8) presented with early-onset hypotonia and global developmental impairment. Neonatal hypotonia was present in all patients (8/8). Respiratory insufficiency after birth was observed in 3/8 individuals, including congenital pneumonia in two of them. Congenital apnea was reported in 3/8 patients. Feeding difficulties occurred in 6/8 patients, with one requiring parenteral nutrition. Additional neonatal features included hypothermia (2/8) and hypersomnolence (5/8).

Intellectual disability was diagnosed in 5/8 individuals (two—severe; two—moderate; one—no formal assessment), while the remaining three were too young for formal assessment. Speech delay was present in all patients, with absent speech in 3/8.

Motor development was delayed in all cases; age of achieving independent sitting was available in four patients (P2—18 m, P4—13 m, P5—10 m, P6—4 y); the fifth one, P8, did not achieve this milestone by 19 months of age. Data on the age at independent walking were available in three patients (P2—28 m, P4—23 m, P5—22 m). Notably, the two patients who achieved independent sitting (P6, P7) did not attain independent ambulation.

Epilepsy was diagnosed in 5/7 patients. In four cases (P3, P6, P7, P8) seizure onset was in the first year of life, and in one case (P4), it developed at the age of 13 years. No information was available on the clinical or electrographic diagnosis of epilepsy in patient P1. The clinical presentation of the observed seizures was most consistent with focal to bilateral tonic–clonic epilepsy. EEG recordings in patients with overt epilepsy were abnormal, showing sharp waves and episodes of sharp wave–slow wave complexes, predominantly with focal distribution [26]. Tremor was observed in 2/8 individuals. Brain imaging findings were largely nonspecific; enlarged lateral ventricles were observed in 2/8 patients, and delayed myelination was observed in 1/8.

Additional features included constipation (4/8), gastroesophageal reflux (1/8), drooling (2/8), and laryngeal laxity (1/8). Visual abnormalities were documented in 4/8 patients (4/4), including strabismus (2/4), optic nerve atrophy (1/4), and abnormal visual evoked potentials with nystagmus (1/4). Facial dysmorphism was noted in 5/8 patients and was generally nonspecific, often described as a myopathic facial appearance. Additional features included up-slanting palpebral fissures (2/8) and retrognathia (1/8). Follow-up head circumference measurements were available for four patients (P2—55 cm at 10 y, P4—54 cm at 11 y, P5—47 cm at 7 m, P7—50 cm at 9 y); microcephaly was identified only in patient P7.

Systemic involvement was limited. Cardiac anomalies were present in one patient (patent foramen ovale and ventricular septal defect). Skeletal abnormalities included scoliosis (2/8), high-arched palate (2/8), malocclusion (1/8), and hip dysplasia (1/8). Hypothyroidism was diagnosed in 2/8 patients.

### 3.2. Molecular Findings

The *PURA* pathogenic/likely pathogenic variants identified in our study group of eight clinically diagnosed *PURA*-NDD patients are summarized in Table 2. In total, eight various single-nucleotide variants (SNVs) in heterozygous form were identified in exon 1 of the *PURA* gene. The SNV spectrum comprised five missense and three frameshift variants: two deletions and one indel, respectively. All missense substitutions were located in the Purine-rich element-binding protein family (PUR repeats I-III). Three identified frameshift variants introduced a premature termination codon, leading to truncation of the encoded protein. As a consequence, these alterations are predicted to result in the loss of normal gene function, primarily due to the production of truncated, nonfunctional proteins and reduced functional *PURA* dosage. Only two variants were confirmed de novo, while in six cases, inheritance could not be determined due to missing biparental DNA.

Overall, four of the eight identified variants (50%) were novel, including: c.311T>G p.(Met104Arg), c.406_407del p.(Gln136Glyfs*64), c.515A>C p.(Gln172Pro), and c.885delinsGC p.(His296Profs*21).

Among the four known variants, two missense changes, p.(Ala89Pro) and p.(Phe231Cys), and one frameshift deletion, p.(Val226Glyfs*67), have been previously described in the literature in patients with the *PURA*-NDD phenotype [2,8,27]. The remaining known missense variant, p.(Asn152Ile), has been submitted in the ClinVar database (accession: VCV001695143.27).

## 4. Discussion

In this study, we present the clinical characteristics of eight individuals with *PURA*-related neurodevelopmental disorder, further expanding the phenotypic spectrum associated with pathogenic variants in the *PURA* gene. Consistent with previous reports, the clinical presentation in our cohort was dominated by neonatal hypotonia, global developmental delay, speech impairment, and delayed motor milestone acquisition, supporting the view that PURA syndrome represents a severe neurodevelopmental condition with early onset [3,6,7,10,13,14,23].

Neonatal manifestations were highly prevalent in our cohort. All patients presented with hypotonia beginning in the neonatal period, confirming this feature as one of the most characteristic and earliest clinical signs of the disorder [6,7,12,15,18,19,28]. In addition, respiratory complications, including congenital apnea and respiratory insufficiency, were observed in two out of six patients. Similar findings have been repeatedly described in the literature and likely reflect central nervous system dysfunction affecting respiratory regulation [3,6,8,12,18,29]. Feeding difficulties, which affected most individuals in our cohort, are also a well-recognized component of PURA syndrome and may result from hypotonia, impaired swallowing coordination, and reduced alertness [3,6,7,10,11,18,29]. The coexistence of hypersomnolence and hypothermia in several patients further supports the hypothesis of disturbed autonomic and central nervous system regulation in affected individuals [10,15,28]. Previous experimental studies additionally suggest that *PURA* dysfunction may impair neuronal maturation and posttranscriptional regulation, contributing to the multisystem neurological phenotype [18,20,25,30].

Developmental impairment was universal in our cohort. All patients exhibited global developmental delay, with particularly severe impairment of speech and motor functions [2,3,6,7,9,13,14,23,28,31]. Three patients remained nonverbal, while all individuals demonstrated delayed achievement of motor milestones. The broad range in the age of independent sitting, from 10 months to 4 years, illustrates the marked phenotypic variability characteristic of *PURA*-related disorders. Importantly, the two patients with the most delayed sitting acquisition did not achieve independent walking, suggesting that early motor development may have prognostic value for later functional outcomes, although this observation requires confirmation in larger cohorts [18,31]. Similar variability in motor and cognitive outcomes has been described in larger cohorts and individual case reports [10,11,13,18]. Although intellectual disability was formally diagnosed in only five patients due to the young age of several individuals, cognitive impairment is likely to become evident in most affected patients during longitudinal follow-up [6,11,12,13].

Epilepsy was identified in the majority of patients and demonstrated variable age at onset and electroclinical presentation. While seizures typically begin in infancy in PURA syndrome, one patient in our cohort experienced the first epileptic episode at 13 years of age, highlighting the possibility of delayed epilepsy onset. EEG abnormalities were consistently observed in patients with clinical seizures and predominantly showed focal epileptiform discharges [3,8,9,10,12,15,20]. Epileptic spasms, generalized seizures, and pharmacoresistant epilepsy have also been described in previous reports [3,6,9,10,12,18,28]. Brain magnetic resonance imaging (MRI) findings remained largely nonspecific, as previously reported in the literature [6,12,13,28]. Mild ventriculomegaly and delayed myelination were the only abnormalities detected, supporting the notion that significant structural brain malformations are uncommon in *PURA*-related neurodevelopmental disorder, although isolated reports of megalencephaly and enlarged brainstem structures have been published [4,18].

Additional neurological and systemic manifestations observed in our cohort further illustrate the broad clinical spectrum of the disease. Gastrointestinal problems, particularly constipation, were relatively common and may be secondary to generalized hypotonia and autonomic dysfunction [3,6,12,18,28,29]. Ophthalmological abnormalities were identified in all evaluated patients, including strabismus, optic nerve atrophy, nystagmus, and abnormal visual evoked potentials, emphasizing the importance of routine ophthalmologic assessment in affected individuals [3,12,18]. Facial dysmorphism was present in most patients but remained nonspecific, frequently manifesting as a myopathic facial appearance. This finding is consistent with previous reports describing subtle and variable craniofacial features rather than a recognizable syndromic gestalt [7,9,12,13,18]. Rare phenotypic associations, including cutis laxa-like features and vitiligo, have additionally been reported, further broadening the phenotypic spectrum linked to *PURA* variants [17,31].

Systemic involvement in our cohort appeared to be relatively limited. Congenital heart defects were identified in only one patient, and skeletal abnormalities were generally mild. Nevertheless, scoliosis, hip dysplasia, and craniofacial abnormalities such as high-arched palate and malocclusion may contribute significantly to long-term morbidity and functional impairment [9,13,18]. Interestingly, hypothyroidism was diagnosed in two patients. Although endocrine abnormalities are not among the most commonly reported manifestations of PURA syndrome, this observation may suggest that thyroid dysfunction could represent an underrecognized component of the phenotype and warrants further investigation.

Our findings are broadly consistent with previously published cohorts and reinforce the concept of considerable clinical heterogeneity in *PURA*-related neurodevelopmental disorder [10,11,12,13,18,19]. Despite the relatively small sample size, which reflects the rarity of the condition, this study contributes additional detailed phenotypic data and highlights several clinically relevant observations, including delayed epilepsy onset and possible endocrine involvement. The variability in disease severity observed even within this small cohort underscores the importance of individualized multidisciplinary care and long-term neurological follow-up. The main limitation of our study is the limited cohort size and incomplete longitudinal data for some patients, particularly younger children in whom neurodevelopmental outcomes could not yet be fully assessed. Furthermore, not all patients underwent uniform specialist evaluations, which may have resulted in the underestimation of certain systemic manifestations. Nevertheless, the detailed clinical characterization presented here provides further insight into the natural history and phenotypic variability of *PURA*-related neurodevelopmental disorder.

Molecular analysis identified eight *PURA* variants in eight probands using different high-throughput sequencing approaches (targeted NGS panel and WGS).

All detected variants were located in exon 1, consistent with the single-exon structure of *PURA* [1]. The variants included both missense substitutions and loss-of-function (LoF) alterations such as frameshift deletions and indels. Overall, the results support haploinsufficiency as the primary disease mechanism underlying *PURA*-related neurodevelopmental disorder [5,18].

Missense variants constituted the majority of detected changes and clustered within conserved PUR domains (PUR-bd_fam I–III), which are critical for nucleic acid binding. This domain-specific enrichment highlights the functional importance of these regions [5,24]. Among them, previously reported variants such as p.(Ala89Pro) and p.(Phe231Cys) were classified as likely pathogenic or pathogenic based on ACMG criteria [24,25]. The de novo occurrence of p.(Ala89Pro) further supports its pathogenicity. Novel missense variants p.(Met104Arg) and p.(Gln172Pro) were classified as likely pathogenic based on moderate ACMG evidence, including localization within critical functional domains and in silico predictions. However, the absence of segregation and functional studies limits definitive interpretation. Similarly, the variant p.(Asn152Ile), previously reported as a VUS in ClinVar, was classified as likely pathogenic in this study due to additional supporting evidence (PP4), illustrating the dynamic nature of variant interpretation as new data emerge.

The identified missense variants, including both reported previously and novel pathogenic changes classified according to ACMG criteria, are predicted to impair *PURA* protein function, particularly because they affect conserved PUR domains involved in nucleic acid binding. This interpretation is consistent with the established haploinsufficiency model of *PURA*-NDD. However, in the absence of functional studies, the exact molecular consequences of individual missense variants remain uncertain. Therefore, these variants should be interpreted as likely reducing *PURA* function rather than as definitively proven loss-of-function alleles. Importantly, there is currently no evidence supporting a gain-of-function (GoF) mechanism in *PURA*-related disease, and the phenotypic spectrum associated with both truncating and missense variants appears to be unified by the partial or complete loss of protein function.

A notable proportion (3/8) of the identified variants were typical LoF changes, including p.(Gln136Glyfs64), p.(Val226Glyfs67), and p.(His296Profs*21). These variants introduce premature termination codons and are fully consistent with a loss-of-function mechanism, as reflected in high ACMG scoring (PVS1). Two of these variants (in P6 and P8) were classified as pathogenic, with P8 additionally confirmed as de novo, further strengthening its clinical relevance. These findings are consistent with previous reports demonstrating that truncating variants in *PURA* have been associated with severe neurodevelopmental phenotypes [3,5,18,18].

Most variants lacked confirmed inheritance information, although two de novo events (P1 and P8) provide strong evidence supporting pathogenicity in neurodevelopmental disorders. The occurrence of de novo variants is consistent with the known genetic architecture of *PURA*-related conditions [5,6,30].

From a methodological perspective, the use of comprehensive molecular testing (NGS panel and WGS) proved complementary, enabling the detection of both single-nucleotide variants and gross insertions and deletions. The rationale for using a targeted NGS panel in seven patients instead of whole-exome/genome sequencing was based on its intended clinical application. As a first-line diagnostic approach, the panel offers substantially lower costs, shorter turnaround times, and more straightforward data interpretation compared with WES or WGS, while maintaining a high diagnostic yield. In the CMHI pediatric cohort, a definitive molecular diagnosis was established in approximately 50% of patients with different genetic rare diseases, demonstrating the effectiveness of this approach in routine clinical practice. Consequently, the use of the targeted panel substantially reduces the number of patients requiring more comprehensive and considerably more expensive analyses, such as WES/WGS.

A limitation of the applied NGS panel (used in most patients in this study) compared with WGS is the inability to identify variants located outside the regions covered by the panel design, particularly in deep intronic regions and regulatory elements. In our seven patients, the presence of additional potentially relevant variants in genes not included in the panel cannot be excluded, and such variants may contribute to the observed phenotypic variability while remaining undetected due to the restricted genomic scope of the assay. Consequently, this limitation may result in failure to detect a subset of pathogenic genetic alterations. Nevertheless, the high diagnostic yield and lower cost of the assay support the use of the panel as an effective diagnostic tool for first-line testing.

In general, a broad genomic approach facilitated a genotype-first diagnostic strategy, as the clinical presentation was not sufficiently specific to guide a targeted differential diagnosis. Consequently, molecular findings in the *PURA* gene were essential for establishing the final diagnosis, underscoring the key role of unbiased genomic testing in the evaluation of phenotypically heterogeneous neurodevelopmental disorders. No clear genotype–phenotype correlations were identified in our cohort, which is consistent with previous reports and likely reflects the limited sample size and clinical heterogeneity of *PURA*-NDD.

## 5. Conclusions

In conclusion, our cohort confirms that *PURA*-related neurodevelopmental disorder is characterized by early-onset hypotonia, severe developmental impairment, and frequent epilepsy, accompanied by variable respiratory, gastrointestinal, ophthalmologic, and skeletal manifestations. The recognition of the characteristic neonatal features may facilitate earlier diagnosis and the implementation of supportive multidisciplinary management. Further studies involving larger cohorts and long-term follow-up are necessary to better define genotype–phenotype correlations and the full clinical spectrum of this rare disorder.

Our results expand also the molecular profile of *PURA* by reporting both novel and known pathogenic variants affecting critical functional domains. These findings reinforce haploinsufficiency as the central disease mechanism and emphasize the importance of integrating clinical, bioinformatic, and genetic evidence in variant classification. Further functional studies and the continued accumulation of genotype–phenotype correlations will be essential to refine the interpretation of missense variants and improve diagnostic accuracy in *PURA*-related neurodevelopmental disorders.

*PURA*-related neurodevelopmental disorder, caused by pathogenic variants in the *PURA*, represents a paradigmatic example of how next-generation sequencing has reshaped the discovery and characterization of rare genetic conditions. First delineated in 2014, this disorder is primarily associated with de novo variants, leading to haploinsufficiency of the Pur-alpha protein, a highly conserved nucleic acid–binding protein involved in DNA replication, transcriptional regulation, and neuronal mRNA transport. The disruption of these processes results in impaired neuronal development and synaptic function. Clinically, PURA syndrome is characterized by neonatal hypotonia, severe developmental delay, absent or minimal speech, feeding difficulties, and variable occurrence of epilepsy and respiratory abnormalities. Despite some phenotypic overlap with other neurodevelopmental syndromes such as Rett, Angelman, Pitt–Hopkins, and other metabolic syndromes, *PURA* disease has emerged as a distinct clinical entity with a recognizable spectrum. The “mutational” landscape includes nonsense, frameshift, missense variants, and gross deletions, with limited but growing evidence for genotype–phenotype correlations. Advances in whole-exome and whole-genome sequencing have significantly improved diagnostic yield, particularly in patients with previously unexplained encephalopathies. Current management remains supportive; however, ongoing research into *PURA* function and RNA biology is opening potential avenues for targeted therapeutic strategies. As such, PURA disease exemplifies the integration of genomic technologies, molecular biology, and clinical phenotyping in advancing the understanding of rare disorders.

## Figures and Tables

**Table 1:** Clinical characteristics of the patients.

	P1	P2	P3	P4	P5	P6	P7	P8
**Age at last follow-up**	17 m	11 y	11 y	15.5 y	5 y	14 y	12 y	19 m
**Sex**	M	M	M	F	M	F	F	F
***PURA* variant (NM_005859.5)**	c.c.265G>C p.(Ala89Pro)	c.311T>G p.(Met104Arg)	c.406_407del p.(Gln136Glyfs*64)	c.455A>T p.(Asn152Ile)	c.515A>C p.(Gln172Pro)	c.675_676del p.(Val226Glyfs*67)	c.692T>G p.(Phe231Cys)	c.885delinsGC p.(His296Profs*21)
**Birth weight**	3835 g P80	2950 g P20		3440 g P75	3740 g P60	3000 g P25	3330 g P50	3660 g P75
**Birth length**		55 cm P50		55 cm P50	58 cm P90	56 cm P75	58 cm P97	57 cm P90
**Apgar score (5 min)**	10	10		9	10	10	10	7–8
**Neonatal hypotonia**	+	+	+	+	+	+	+	+
**Respiratory insufficiency**	+	+ (congenital pneumonia)	-	-	-	-		+ (congenital pneumonia, pneumothorax)
**Feeding difficulties**	+	+	-	+	+	-	+	+ (parenteral nutrition for 9 days at 1 m)
**Hypothermia**	-	-	-	-	-	+	-	+
**Hypersomnolence**	+	-	-	+	+	+	-	+
**Congenital apnea**	+	-	-	+	-	-	-	-
**Developmental delay**	+	+	+	+	+	+	+	+
**Intellectual disability**		Moderate	+	Moderate		Severe	Severe	
**Delayed speech**	+	Not yet achieved	+	3 y	Yes, simple sentences at 5 y	Not yet achieved	Not yet achieved	+
**Delayed sitting**	+	18 m	+	13 m	10 m	4 y	+	Not yet achieved
**Delayed walking**	+	28 m	+	23 m	22 m	Not yet achieved	At 9 y moves on buttocks	Not yet achieved
**Epilepsy**		-	+	+ (13 y)	-	+	+	+
**EEG**	Normal	Normal		Normal		Sharp waves in right parietal region	Numerous sharp waves and episodes of sharp wave–slow wave in parietal and occipital regions	Sporadic localized changes
**Tremor/Dystonia**	-	-	-	-	-	+	-	+
**Vision problems**		Convergent strabismus			Divergent strabismus	Optic disk atrophy		VEP abnormal, nystagmus
**Facial dysmorphia**	Coarse face	Myopatic face, up-slanting palpebral fissures, microstomia		Myopatic face, up-slanting palpebral fissures, short nose, thin upper lip, retrognathia		Myopatic face	Myopatic face, deep-set eyes	Myopatic face
**OFC (age)**		55 cm (10 y) P75		54 cm (11 y) P60	47 cm (7 m) P97		50 cm (9 y) P3	
**Systemic features**	PFO, VSD	Hypospadiasis, high-arched palate, malocclusion	Hypothyroidism	Hypothyroidism, scoliosis, high-arched palate, crowded teeth	Flat feet	Scoliosis, high-arched palate	Hip dysplasia, right hip surgery at 10 y	
**Brain** **magnetic resonance imaging**	Enlarged lateral ventricles (2 m)					Delayed myelination (2 y)	Enlarged left lateral ventricle (9 y)	
**Other**	Laryngeal laxity	Drooling, overweight		Constipation, hiccup, drooling	Constipation, aggression	Constipation	GI reflux, constipation	NEC, lack of eye contact

Abbreviations: GI—gastrointestinal, NEC—necrotising enterocolitis, P—percentile.

**Table 2:** Characteristics of molecular variants identified in the *PURA* gene.

Patient ID	Genetic Testing Method in Proband	Nucleotide Change (NM_005859.5)	Amino Acid Change (NP_005850.1)	Exon (Intron)	Variant Type	Variant Effect	Protein location/Affected Domain	Reported Status	Inheritance	Pathogenicity Status in Databases			ACMG/AMP and ACGS Variant Classification										
										ClinVar^#^	LOVD	HGMD	PVS1	PM1	PM2	PM5	PM6	PP1	PP3	PP4	PP5	Points	Verdict
P1	NGS P1000	c.265G>C	p.(Ala89Pro)	1	SNV	missense substitution	PUR-bd_fam (I)	known	de novo	P	n/a	DM	0	2	1	0	2	0	2	2	1	10	LP
P2	NGS P1000	c.311T>G	p.(Met104Arg)	1	SNV	missense substitution	PUR-bd_fam (I)	novel	unknown	n/a	n/a	n/a	0	2	1	0	0	0	2	2	0	7	LP
P3	WGS	c.406_407del	p.(Gln136Glyfs*64)	1	SNV	frameshift deletion	null	novel	unknown	n/a	n/a	n/a	8	0	1	0	0	0	0	2	0	11	LP
P4	NGS P1000	c.455A>T	p.(Asn152Ile)	1	SNV	missense substitution	PUR-bd_fam (II)	known	unknown	VUS	n/a	n/a	0	2	1	0	0	0	2	3	0	8	LP
P5	NGS P1000	c.515A>C	p.(Gln172Pro)	1	SNV	missense substitution	PUR-bd_fam (II)	novel	unknown	n/a	n/a	n/a	0	2	1	0	0	0	2	1	0	6	LP
P6	NGS P1000	c.675_676del	p.(Val226Glyfs*67)	1	SNV	frameshift deletion	null	known	unknown	P	n/a	DM	8	0	1	0	0	0	1	3	2	15	P
P7	NGS P1000	c.692T>G	p.(Phe231Cys)	1	SNV	missense substitution	PUR-bd_fam (III)	known	unknown	P	n/a	DM	0	2	1	2	0	1	2	3	1	12	P
P8	NGS P1000	c.885delinsGC	p.(His296Profs*21)	1	SNV	frameshift indel	null	novel	de novo	n/a	n/a	n/a	8	0	1	0	2	0	0	3	0	14	P

n/a, not available; SNV, single-nucleotide variant; Protein location (affected domain): PUR-bd_fam, Purine-rich element binding protein family (PUR repeats I–III); null, complete loss of protein; P, Pathogenic (≥10 points); LP, likely pathogenic (6–9 points); ACMG/AMP (the American College of Medical Genetics and Genomics and the Association for Molecular Pathology) and ACGS (the Association for Clinical Genomic Science criteria [7,23]; PVS, Pathogenic Very strong; PS, Pathogenic Strong; PM, Pathogenic Moderate; PS, Pathogenic Supporting). NGS P1000: the original Children’s Memorial Health Institute NGS panel of >1000 clinically relevant genes (details in Supplementary Table S1). The novelty of the identified variants was assessed using ClinVar (https://www.ncbi.nlm.nih.gov/clinvar, accessed on 4 May 2026) and stars (0 to 4) to provide a graphical representation of the aggregate review status, as well as the LOVD (the Leiden Open Variation Database, https://www.lovd.nl/, accessed on 4 May 2026) and HGMD (the Human Gene Mutation Database, www.hgmd.cf.ac.uk, accessed on 4 May 2026) databases, and to see if the known related clinical condition had been confirmed. The nomenclature of molecular variants follows the Human Genome Variation Society (HGVS) guidelines (https://hgvs-nomenclature.org/) and refers to the Matched Annotation from the NCBI and EMBL-EBI (MANE) Select transcript, which aligns with the GRCh38/hg38 reference genome assembly.

## Data Availability

The original contributions presented in this study are included in the article. Further inquiries can be directed to the corresponding author.

## Supplementary Materials

The following supporting information can be downloaded at: https://www.mdpi.com/article/10.3390/genes17070765/s1, Supplementary Table S1. The original Children’s Memorial Health Institute NGS panel of >1000 clinically relevant genes.

## Abbreviations

The following abbreviations are used in this manuscript:

ACGS	Association for Clinical Genomic Science
ACMG	American College of Medical Genetics and Genomics
AMP	Association for Molecular Pathology
CMHI	Children’s Memorial Health Institute
HGMD	Human Gene Mutation Database
HGVS	Human Genome Variation Society
LP	Likely pathogenic
LOVD	Leiden Open Variation Database
NGS	Next-generation sequencing
Null	Complete loss of protein
P	Pathogenic
PM	Pathogenic moderate
POLdb	Polish rare disease database
PP	Pathogenic supporting
PVS	Pathogenic very strong
PUR-bd_fam	Purine-rich element binding protein family (PUR repeats I-III)
SNV	Single-nucleotide variant
